# Road traffic accidents and posttraumatic stress disorder in an orthopedic setting in south-eastern Nigeria: a controlled study

**DOI:** 10.1186/1757-7241-19-39

**Published:** 2011-06-22

**Authors:** Obiora Iteke, Muideen O Bakare, Ahamefule O Agomoh, Richard Uwakwe, Jojo U Onwukwe

**Affiliations:** 1 General Adult Psychiatry Unit, Federal Neuropsychiatric Hospital, New Haven, Enugu, Enugu State, Nigeria; 2 Child and Adolescent Unit, Federal Neuropsychiatric Hospital, New Haven, Enugu, Enugu State, Nigeria; 3 Forensic Psychiatry Unit, Federal Neuropsychiatric Hospital, New Haven, Enugu, Enugu State, Nigeria; 4 Department of Mental Health, Nnamdi Azikiwe University Teaching Hospital, Nnewi, Anambra State, Nigeria; 5 Community Psychiatry Unit, Federal Neuropsychiatric Hospital, New Haven, Enugu, Enugu State, Nigeria

## Abstract

**Background:**

Psychiatric liaison services are rare in trauma units of various hospitals in Nigeria and other sub-Saharan African countries. The occurrence of road traffic accidents (RTAs) resulting from low standard of road construction and inadequate maintenance have been on the increase in Nigeria. While the physical consequences of such RTAs are obvious, the psychological consequences are often not apparent. This study assessed the prevalence of posttraumatic stress disorder (PTSD) among victims of RTAs and compared same with controls drawn from a population who have not experienced RTAs. It also assessed the associated socio-demographic variables.

**Method:**

Study population consisted of one hundred and fifty RTA victims and two different control groups drawn from the population consisting of staffs of Federal Neuropsychiatric Hospital, Enugu, Nigeria and that of National Orthopedic Hospital, Enugu, Nigeria, 150 people in each control group were matched for age and sex with the RTA victims and they were interviewed with PTSD module of Mini International Neuropsychiatric Interview (MINI) and their socio-demographic variables obtained with socio-demographic questionnaire.

**Results:**

The prevalence of PTSD among RTA victims and the two control groups were 26.7%, 8.0% and 8.7% respectively. The difference in prevalence was statistically significant with RTA victims more likely to experience PTSD compared to the two control groups (X^2 ^= 27.23, df = 2, p = 0.001). Gender influenced the prevalence of PTSD among victims of RTAs and the controls, with females more likely to experience PTSD when compared to the males. Among victims of RTAs, being gainfully employed prior to the accidents increased the likelihood of developing PTSD and this was statistically significant (X^2 ^= 20.09, df = 1, p = 0.000).

**Conclusions:**

There is urgent need to pay more attention to developing consultation-liaison psychiatry services in trauma units of Nigerian hospitals, including orthopedic hospitals located in different geographical zones of the country.

## Background

Developing countries, of which sub-Saharan African countries are inclusive, have been known to face double burden of diseases [1,2]. Poor governance and corrupt practices in most sub-Saharan African countries had led to poor standards in construction and maintenance of social amenities like roads amongst others. There have been many cases of road traffic accidents (RTAs) resulting from low construction standards and poor road maintenance [3,4].

Avoidable road traffic accidents among other accidents have continued to add to morbidity and mortality in most sub-Saharan African countries. It appears like most sub-Saharan African countries are then experiencing another disease burden from the preventable accidents resulting from poor road infrastructure. It may therefore not be out of place to talk of a triple burden of disease. The issue of road traffic accidents is reaching an alarming state in Nigeria where Federal Road Safety Corps (FRSC) has continued to report increased prevalence in road traffic accidents on a yearly basis [4].

The inclusion of motorcycles in Nigeria as a means of commercial transportation has added to the likelihood occurrence of these road traffic accidents (RTAs) [5]. Frequent injuries sustained in most of the reported RTAs included bone fractures, which are often treated in specialized orthopedic hospitals in this environment.

While the physical consequences of these RTAs are apparent in soft tissue injuries and bone fractures [5], psychological consequences such as PTSD amongst others are not easily obvious. Less attention has therefore been paid to the psychological consequences of RTAs in Nigeria where the prevalence has been reported to be on the increase [4,5]. While many literatures from other parts of the world had addressed issue of posttraumatic stress disorder complicating road traffic accidents and other traumas, such information are largely unavailable in sub-Saharan Africa. In other parts of the world, the prevalence of posttraumatic stress disorder after road traffic accidents range between 8.5% and 39% [6-9]. There had been inconsistent report on socio-demographic factors associated with developing posttraumatic stress disorder following traumatic events. Only factor of gender appeared to have been consistent in most reports, with female gender often more prone to developing symptoms of posttraumatic stress disorder after traumatic events [6-9].

Despite the potential psychological consequences of road traffic accidents, liaison psychiatry services are rare practices in various orthopedic hospitals and trauma units of other hospitals in Nigeria.

This study therefore assessed the prevalence of PTSD among injured victims of RTAs in an orthopedic setting in South Eastern Nigeria and compared same to matched group of individuals from the general population, randomly chosen from staffs of Federal Neuropsychiaric Hosital, Enugu, Nigeria and that of National Orthopedic Hospital, Enugu, Nigeria. It also assessed associations between PTSD and various socio-demographic variables.

## Methods

The location of the study was the out-patient unit of National Orthopedic Hospital Enugu (NOHE), Nigeria. This hospital, which was established in 1975, has a total of ten wards. The bed capacity of the hospital is about two hundred and twenty (220). The hospital provides specialist medical care to patients in South Eastern States of Nigeria.

Permission to conduct the study was obtained from the Institutional Review Board (IRB) of Federal Neuropsychiatric Hospital, Enugu, (FNHE), Nigeria and the Research, Education and Training (RET) unit of National Orthopedic Hospital, Enugu, (NOHE), Nigeria. Those identified with PTSD were referred to Federal Neuropsychiatric Hospital Enugu, (FNHE), Nigeria for further evaluation and management.

### Sample population

The study population consisted of 150 injured victims of RTAs, aged 18 years and above who were attending the out-patient unit of the National Orthopedic Hospital Enugu (NOHE), Nigeria for follow up treatment from September to December 2008. Consecutive injured RTA victims who gave informed consent were interviewed. Patients with history of head injury during the RTAs were excluded from the study.

The controls were selected from staffs of National Orthopedic Hospital, Enugu (NOHE), Nigeria and Federal Neuropsychiatric Hospital Enugu (FNHE), Nigeria. The two hospitals are located within Enugu metropolis. The controls had not been involved in RTAs.

Because the staffs of National Orthopedic Hospital, Enugu (NOHE), Nigeria were more prone to witnessing injured victims of RTAs being brought to the hospital, it was hypothesized that they could also be prone to developing post-traumatic syndrome and stress disorder. For this reason, a second control group was considered for the study. Thus, 150 individuals from staffs of Federal Neuropsychiatric Hospital, Enugu (FNHE), Nigeria and 150 individuals from staffs of National Orthopedic Hospital, Enugu (NOHE), Nigeria served as control groups 1 and 2 respectively. Therefore, the total population studied was 450 people.

The control subjects were selected randomly from list of staffs of National Orthopedic Hospital, Enugu (NOHE), Nigeria and Federal Neuropsychiatric Hospital Enugu (FNHE), Nigeria. They were matched for age and sex with the study group.

## Materials

### Socio-demographic Questionnaire

A sociodemographic questionnaire was used to elicit such variables as age, sex, marital status, religion, educational level, occupation and the time since the road traffic accidents occurred among the study subjects.

### Mini International Neuropsychiatric Interview (M.I.N.I)[10]

This is a short structured interviewer administered diagnostic instrument, developed for assessing psychiatric disorders based on DSM-IV and ICD-10 diagnostic criteria [10].

### Procedure

Consecutive outpatient attendees who are injured victims of RTA whose accidents occurred not earlier than one month and not later than twelve months before the study were interviewed until the sample size was met.

The outset exclusion criteria included, patients who did not consent to the study, patients who sustained head injury following the RTAs and patients whose RTAs had not lasted for more than one month. However, none of the patients approached for interview refused to give consent. Patients with head injury were excluded because they could develop neurological complications that might introduce bias into the outcome of the study. This group of patients with head injury constituted less than ten percent of the total out-patients population.

Consecutive out-patients attendees that met the inclusion criteria for the study were administered socio-demographic questionnaire to elicit variables such as age, sex, marital status, educational level, occupation and time when the RTA occurred.

As all subjects studied were fluent in English language, they were interviewed with the English version of PTSD module of the Mini International Neuropsychiatric Interview (M.I.N.I) [10] to make a diagnosis of PTSD. The control subjects matched for age and sex to the study subjects were also interviewed with the PTSD module of the M.I.N.I instrument.

### Data analysis

The data were analyzed using the Statistical Package for Social Sciences (SPSS), version 16. Prevalence of posttraumatic stress disorder in each group was determined. Analysis of variance (ANOVA) and chi-square statistics was used to compare the three groups studied on socio-demographic variables. Chi-square statistics was used to test association between presence of posttraumatic stress disorder and socio-demographic variables.

## Results

There were four hundred and fifty (450) participants interviewed. One hundred and fifty (150) injured victims of road traffic accidents, one hundred and fifty (150) participants in the first control group and one hundred and fifty (150) participants in the second control group selected from staffs of FNHE and NOHE, Nigeria who had not been involved in RTAs.

### Socio-demographic variables of the victims of road traffic accidents and the two control groups

#### Age

The age range of the victims of road traffic accidents was 18 to 57 years, and the mean age was 31.61 ± 9.18 years.

The first control group had an age range of 18 to 56 years with a mean age of 32.14 ± 8.85 years, while the second control group had an age range of 19 to 57 years with a mean age of 33.01 ± 8.95 years.

#### Gender

The victims of road traffic accidents consisted of 108(72.0%) males and 42(28.0%) females. The first control group consisted of 108(72.0%) males and 42(28.0%) females, while the second control group consisted of 108(72.0%) males and 42(28.0%) females.

#### Marital status

Fifty-nine (39.3%) of the injured victims of road traffic accidents were married while 91(60.7%) were single. In the first control group, 85(56.7%) were married, 64(42.7%) were single and 1(0.7%) was widowed. For the second control group 95(63.3%) were married and 55(36.7%) were single.

#### Religion

All of the victims of road traffic accidents and the two control groups were Christians.

#### Level of education

Educational attainment was grouped into those with tertiary education and those with below tertiary education. Levels of education in Nigeria are divided into primary, secondary and tertiary. Below tertiary education as used here referred to secondary school education and below, while tertiary education as used here referred to post-secondary school education. One hundred (66.7%) of the injured victims of road traffic accidents had educational level below tertiary, while 50(33.3%) had tertiary education.

In the first control group, 45(30.0%) participants had below tertiary education while 105(70.0%) attained tertiary level of education. For the second control group, 39(26.0%) had below tertiary education while 111(74.0%) had tertiary education.

#### Employment status

Among the victims of road traffic accidents, 89(59.3%) were employed while 61(40.7%) were unemployed. Participants in both the first and second control groups were all employed.

#### Comparison of the three groups on the socio-demographic variables

Using one-way analysis of variance (ANOVA), there was no statistical significant difference in the mean age of the RTA victims and the control groups (F-ratio = 0.92, p = 0.400). There was also no statistical significant difference in sex distribution among the three groups of RTA victims and the control groups (X^2 ^= 0.00, df = 2, p = 1.000). The samples were however different in the distribution of their marital status with those in the control groups being more married than the victims of RTA (X^2 ^= 20.70, df = 4, p = 0.000). They were also different in the distribution of their level of education with those in the control groups attaining tertiary level of education compared to the victims of RTA (X^2 ^= 62.36, df = 2, p = 0.000). All the participants in the control groups were employed. Table 1 showed the socio-demographic variables of the RTA victims and the control groups.

**Table 1:** Socio-demographic variables of the victims of road traffic accidents and the two control groups

Socio-demographic variables	Road Traffic Accident Victims N(%)	Control Group 1 (FNHE staffs) N (%)	Control Group 2(NOHE staffs) N (%)	Statistics
Mean age	31.61 ± 9.18	32.14 ± 8.85	33.01 ± 8.95	F = 0.918 p = 0.400

**Gender:**				
Male	108(72.0%)	108(72.0%)	108(72.0%)	X^2^= 0.000 df = 2
Female	42(28.0%)	42(28.0%)	42(28.0%)	p = 1.000

**Marital Status:**				
Married	59(39.3%)	85(56.7%)	95(63.3%)	X^2 ^= 20.698
Single	91(60.7%)	64(42.7%)	55(36.7%)	df = 4
Widowed	0(0.0%)	1(0.7%)	0(0.0%)	p = 0.000

**Religion:**				
Christianity	150(100.0%)	150(100.0%)	150(100.0%)	

**Level of Education:**				
Below tertiary	100(66.7%)	45(30.0%)	39(26.0%)	X^2 ^= 62.355 df = 2
Tertiary	50(33.3%)	105(70.0%)	111(74.0%)	p = 0.000

**Employment Status:**				
Employed	89(59.3%)	150(100.0%)	150(100.0%)	
Unemployed	61(40.7%)	0(0.0%)	0(0.0%)	

**FNHE **- Federal Neuropsychiatric Hospital, Enugu, Nigeria

**NOHE **- National Orthopedic Hospital, Enugu, Nigeria

#### Prevalence and distribution of PTSD among victims of road traffic accidents and the controls

Among the 150 road traffic accident victims interviewed, forty (26.7%) experienced PTSD while 110(73.3%) did not experience PTSD. In the first control group from the general population (i.e. Staffs of Federal Neuropsychiatric Hospital Enugu, Nigeria) 12(8.0%) experienced PTSD while 138(92.0%) did not experience PTSD. For the second control group from the general population (i.e. Staffs of National Orthopedic Hospital Enugu, Nigeria) 13(8.7%) experienced PTSD while 137(91.3%) did not experience PTSD. The victims of RTA were more likely to experience PTSD compared to people in either of the two control groups. (X^2 ^= 27.23, df = 2, p = 0.001). See Table 1.

### Socio-demographic variables and prevalence of PTSD among victims of RTA and the controls

#### Gender

Among the victims of RTA, 16 females which represented 38.0% of the total female population in this group experienced PTSD when compared to 24 males that represented 22.2% of the male population. The difference in prevalence of PTSD was statistically significant with females being more likely to experience PTSD (X^2 ^= 3.74, df = 1, p = 0.05). See Table 1.

In the first control group, 6 females which represented 14.3% of the female population experienced PTSD, while 6 males which represented 5.6% of the male population experienced PTSD. More females compared to males in this group were likely to experience PTSD, but the difference was not statistically significant (X^2 ^= 2.84, df = 1, p = 0.09).

In the second control group, 7 females which represented 16.7% of the female population experienced PTSD, while 6 males that represented 5.6% of the male population experienced PTSD. The difference was statistically significant with females being more likely to experience PTSD when compared to males (X^2 ^= 4.24, df = 1, p = 0.04). See Table 1.

#### Employment status

Out of a total of 40 individuals (26.7%) who had PTSD among the victims of RTA, 35 individuals who represented 23.3% prevalence of PTSD were gainfully employed prior to the accidents, while 5 individuals who represented 3.3% prevalence of PTSD were unemployed. The difference was statistically significant with those who were employed more likely to experience PTSD compared to those who were unemployed (X^2 ^= 20.09, df = 1, p = 0.000). Gainfully employed prior to the RTAs was defined by the individual concerned having a source of income and independent; either being self employed, employed by the government or private establishments. The two control groups could not be comparatively compared on this demographic variable because individuals in the two control groups were staffs of two government hospitals in Enugu metropolis, south-eastern Nigeria (FNHE and NOHE). See Table 1.

#### Other socio-demographic variables

Other socio-demographic variables such as age, marital status and level of education did not show significant influence on the prevalence of PTSD among the victims of RTA and the two control groups.

## Discussion

The findings of this study revealed that injured road traffic accident victims had higher prevalence of PTSD compared to the two control groups who did not experience RTA.

Female victims of road traffic accidents experienced PTSD more than the male victims of road traffic accidents. Those who were employed among the victims of road traffic accidents experienced PTSD more than those who were not employed. In the first control group from the general population, females experienced PTSD more than the males. In the second control group from the general population, females also experienced PTSD more than the males.

The prevalence of PTSD among the victims of RTAs in this study was found to be 26.7%. This compares with that of some previous study carried out in orthopedic hospitals [11,12]. The findings also agreed with that of Shalev et al [13] who reported a PTSD prevalence of about thirty percent at one month, about seventeen percent at four months among trauma victims.

In the first control group, there was 8.0% prevalence of PTSD while in the second control group it was 8.7%. These findings are consistent with previous reports of PTSD prevalence among United States of America general population in which Breslau et al [14] and Kessler et al [15] reported a PTSD prevalence of eight to nine percent.

Among the victims of RTA and the control groups, there was consistent finding of females experiencing PTSD more than the males. This agreed with previous studies by Breslau et al [14] and Resnick et al [16]. It had been reported that men and women have different ways of responding to danger and expressing distress [17]. During traumatic events, females use dissociative defense mechanism more than males [18]. Females and males also have different self-schemas and world-schemas following exposure to similar traumatic events [19]. The findings that women have more negative self-schemas and world-schemas may be consistent with the increased diagnosis of PTSD in the women [20].

In this study, those who had gainful employment before the RTA experienced PTSD more than the unemployed. This agrees with the reports of another study of RTA victims in which it was found that PTSD scores on Structured Clinical Interview for DSM-IV diagnosis (SCID) were significantly and positively associated with loss of job activity due to the accident [21]. Disruption of business and job activities with probable attendant financial difficulties among the injured victims of RTAs may have led to catastrophic appraisal of the future. Post-trauma factors like financial difficulties have been reported to be independently associated with symptoms of posttraumatic stress disorder [22]. This is more important in the environment of Sub-Saharan Africa where patronage for insurance is very limited.

## Conclusions

The findings of this study point to the need for promoting development of consultation-liaison psychiatry services in trauma units of Nigerian hospitals, including orthopedic hospitals in different geographical zones of the country. Government policies aimed at improving standards of road construction and maintenance in order to forestall avoidable road traffic accidents are advocated in sub-Saharan African sub-region.

## Competing interests

The authors declare that they have no competing interests.

## Authors' contributions

All authors were involved in the conception of the study. OI participated in data collection and wrote the initial draft of the Manuscript. OI, MOB and AOA analyzed the data. All authors were involved in revision of the Manuscript. All authors read and approved the final draft of the Manuscript.
